# The E3 Ubiquitin Ligase Triad3A Negatively Regulates the RIG-I/MAVS Signaling Pathway by Targeting TRAF3 for Degradation

**DOI:** 10.1371/journal.ppat.1000650

**Published:** 2009-11-06

**Authors:** Peyman Nakhaei, Thibault Mesplede, Mayra Solis, Qiang Sun, Tiejun Zhao, Long Yang, Tsung-Hsien Chuang, Carl F. Ware, Rongtuan Lin, John Hiscott

**Affiliations:** 1 The Terry Fox Molecular Oncology Group, Lady Davis Institute for Medical Research, Montreal, Quebec, Canada; 2 Department of Microbiology & Immunology, McGill University, Montreal, Quebec, Canada; 3 Department of Medicine, McGill University, Montreal, Quebec, Canada; 4 Department of Immunology, The Scripps Research Institute, La Jolla, California, United States of America; 5 The Division of Molecular Immunology, La Jolla Institute for Allergy and Immunology, San Diego, California, United States of America; University of Washington, United States of America

## Abstract

The primary role of the innate immune response is to limit the spread of infectious pathogens, with activation of Toll-like receptor (TLR) and RIG-like receptor (RLR) pathways resulting in a pro-inflammatory response required to combat infection. Limiting the activation of these signaling pathways is likewise essential to prevent tissue injury in the host. Triad3A is an E3 ubiquitin ligase that interacts with several components of TLR signaling and modulates TLR activity. In the present study, we demonstrate that Triad3A negatively regulates the RIG-I RNA sensing pathway through Lys^48^-linked, ubiquitin-mediated degradation of the tumor necrosis factor receptor-associated factor 3 (TRAF3) adapter. Triad3A was induced following dsRNA exposure or virus infection and decreased TRAF3 levels in a dose-dependent manner; moreover, Triad3A expression blocked IRF-3 activation by Ser-396 phosphorylation and inhibited the expression of type 1 interferon and antiviral genes. Lys^48^-linked ubiquitination of TRAF3 by Triad3A increased TRAF3 turnover, whereas reduction of Triad3A expression by stable shRNA expression correlated with an increase in TRAF3 protein expression and enhancement of the antiviral response following VSV or Sendai virus infection. Triad3A and TRAF3 physically interacted together, and TRAF3 residues Y440 and Q442—previously shown to be important for association with the MAVS adapter—were also critical for Triad3A. Point mutation of the TRAF-Interacting-Motif (TIM) of Triad3A abrogated its ability to interact with TRAF3 and modulate RIG-I signaling. TRAF3 appears to undergo sequential ubiquitin “immuno-editing” following virus infection that is crucial for regulation of RIG-I-dependent signaling to the antiviral response. Thus, Triad3A represents a versatile E3 ubiquitin ligase that negatively regulates RIG-like receptor signaling by targeting TRAF3 for degradation following RNA virus infection.

## Author Summary

RNA virus infection is detected through TLR-dependent and TLR-independent mechanisms. Early viral replicative intermediates are detected by two recently characterized cystolic viral RNA receptors, RIG-I and MDA-5, leading to the production of pro-inflammatory cytokines and type I interferons (IFNs). Dysfunctional responses, either failure to respond or hyper-responsiveness, may lead to both acute and chronic immunodeficiency and inflammatory diseases. Thus, the intensity and duration of RLR signaling must be tightly controlled. One general mechanism by which innate immune receptors and their downstream adapters are regulated involves protein degradation mediated by the ubiquitination pathway. Our study demonstrates that the E3 ubiquitin ligase Triad3A negatively regulates the RIG-I-like receptor pathway by targeting the adapter molecule TRAF3 for proteasomal degradation through Lys^48^-linked ubiquitin-mediated degradation. Thus, Triad3A represents a key molecule involved in the negative regulation of the host antiviral response triggered by RNA virus infection.

## Introduction

Upon recognition of specific molecular components of viruses, the host cell activates multiple signaling cascades that stimulate an innate antiviral response, resulting in the disruption of viral replication, and the mobilization of the adaptive arm of the immune system. Central to the host antiviral response is the production of type 1 interferons (IFNs), a large family of multifunctional immunoregulatory proteins. Multiple Toll like receptor (TLR)-dependent (TLR-3, -4, -7 and 9) and RIG-I-like receptor (RLR) pathways are involved in the cell specific regulation of Type I IFNs, with accumulating evidence that cooperation between different pathways is required to ensure a robust and controlled activation of antiviral response [1],[2],[3]. RIG-I-like receptors (RLRs) - the retinoic acid-inducible gene-I (RIG-I) and melanoma differentiation-associated gene-5 (MDA-5) - are novel cytoplasmic RNA helicases that recognize viral RNA present within the cytoplasm. Although both TLR7 and TLR9 are critical for recognition of viral nucleic acids in the endosomes of plasmacytoid dendritic cells (pDCs), most other cell types recognize viral RNA intermediates through the RLR arm of the innate immune response [4],[5],[6]. Structurally, RIG-I contains two caspase activation and recruitment domains (CARD) at its N-terminus and RNA helicase activity in the C-terminal portion of the molecule [4]. The C-terminal regulatory domain (CTD) (aa 792–925) of RIG-I binds viral RNA in a 5′-triphosphate-dependent manner and activates RIG-I ATPase inducing RNA-dependent dimerization and structural alterations that enable the CARD domain to interact with other downstream adapter protein(s) leading to the transcription of antiviral genes [7],[8],[9].

RIG-I-dependent signaling to the IKKα/β complex and to TBK1/IKKε is transmitted via a CARD domain containing adapter molecule – alternatively named mitochondrial antiviral signaling (MAVS), interferon-β stimulator 1 (IPS-1), virus induced signaling adapter (VISA), CARD adapter inducing IFN-β (CARDIF) [10],[11],[12],[13]. MAVS localizes to the outer mitochondria membrane via a C-terminal mitochondrial transmembrane targeting domain (TM), and its mitochondrial localization acts as a pivotal point for triggering the antiviral cascade via activation of NF-κB and IRF-3 [3],[14],[15],[16].

Activation of TLRs and RLRs results in the dissemination of an antiviral and antimicrobial cascade necessary to combat invading pathogens [17],[18],[19]. Limiting the intensity and duration of TLR and RLR signaling is likewise essential to prevent this protective response from causing inflammatory or autoimmune injury to the host. Ubiquitination is a post-translational modification by which signaling is suppressed in many regulatory pathways [20]. Lys^48^-linked ubiquitination is one of the most common pathways to target proteins for 26S proteasomal degradation [21], whereas Lys^63^-linked ubiquitination is involved in protein-protein interactions, recruitment, and assembly of signaling complexes [22],[23]. It has become clear that ubiquitination of signaling adapters is an integral part of NF-*κ*B and IFN signaling in response to virus pathogen associated molecular patterns (PAMPs). Deubiquitinating enzymes that remove Lys^63^-linked ubiquitin are also emerging as key negative regulators of the IFN and NF-κB pathways [16],[24],[25],[26],[27]. For example, the deubiquitinating enzyme A (DUBA), a novel OTU-domain DUB negatively regulates IFN signaling following RIG-I, MDA5 or TLR3 stimulation [28]. DUBA specifically removes Lys^63^-linked ubiquitin chains from TRAF3, resulting in the disruption of interaction between TRAF3 and the downstream kinases IKKε and TBK1 and subsequent blockade of IRF-3 and IRF-7 phosphorylation [28].

The activation of RIG-I/MDA-5 ultimately leads to the TM-dependent dimerization of the MAVS N-terminal CARD domains, thereby providing an interface for direct binding to and activation of the tumor necrosis factor (TNF) receptor-associated factor (TRAF) family members that are involved in both the IFN and NF-*κ*B arms of the innate immune response [29],[30]. TRAF3 is an adapter molecule that is required for the induction of type I IFN and anti-inflammatory cytokine interleukin-10 (IL-10), but is dispensable for expression of pro-inflammatory cytokines in response to viral infection and TLR ligation in bone marrow-derived macrophages (BMMs), plasmacytoid dendritic cells (pDCs), and murine embryonic fibroblasts (MEFs) [31],[32].

TRAF3 was the first TRAF demonstrated to directly associate with CD40. Subsequently, it was shown that TRAF3 negatively regulates CD40 signaling by competing with TRAF2 for CD40 binding, thus impeding CD40-TRAF2 mediated JNK and NF-κB activation [33]. Crystal structure of the binding crevice of TRAF3 bound in complex with a 24-residue fragment of the cytoplasmic portion of BAFF receptor (BAFF-R), revealed two amino acids in TRAF3 -Y440A and Q442- that are involved in BAFF-R interaction [34]. Interestingly, other TNFRs such as CD40 contain similar TRAF-interacting motifs (TIMs), defined by the consensus sequence PxQx(T/S), that interact with the same binding crevice on TRAF3 [35],[36]. In addition, the TRAF family member–associated NF-κB activator (TANK) adapter and the viral oncogene LMP1 of the Epstein Barr Virus also bind to the same structural crevice of TRAF3 [37],[38]. MAVS regulation of type I IFN induction is achieved by direct and specific interaction with the TIM of TRAF3; interestingly point-mutation of the TIM domain completely abrogates TRAF3-mediated IFN-α production in response to Sendai virus infection [39].

Triad3A is a RING finger type E3 ubiquitin-protein ligase that promotes Lys^48^-linked ubiquitination and proteolytic degradation of TLR4 and TLR9 and negatively regulates their activation by lipopolysaccharide and CpG-DNA, respectively [40]. Triad3A is the most abundant alternatively spliced form of the Triad family. In addition, Triad3A interacts and promotes down-regulation of two TIR domain containing adapter molecules, TIR-domain-containing adapter-inducing IFN-β (TRIF) and TRIF-related adapter molecule (TIRAP). Moreover, Triad3A acts as a negative regulator of TNF-α signaling by interacting with the TIR homologous (TIRH) domain containing protein receptor-interacting protein 1 (RIP1) [41]. This interaction effectively disrupts RIP1 binding to the TNF-R1 complex and impedes RIP-1-mediated NF-*κ*B activation [41].

The identification of a TIM sequence in the N-terminus of Triad3A -using a program written in python language (http://www.biopython.org)- as well as the previously characterized function of Triad3A in TLR signaling, prompted us to investigate the role of Triad3A in the regulation of the RIG-I/MAVS signaling via TRAF3. In the present study, we demonstrate that Triad3A negatively regulates the RIG-I signaling pathway through Lys^48^-linked ubiquitin-mediated degradation of TRAF3, resulting in the inhibition of the type I IFN response.

## Results

### Triad3A disrupts RIG-I signaling

**Figure 1:**
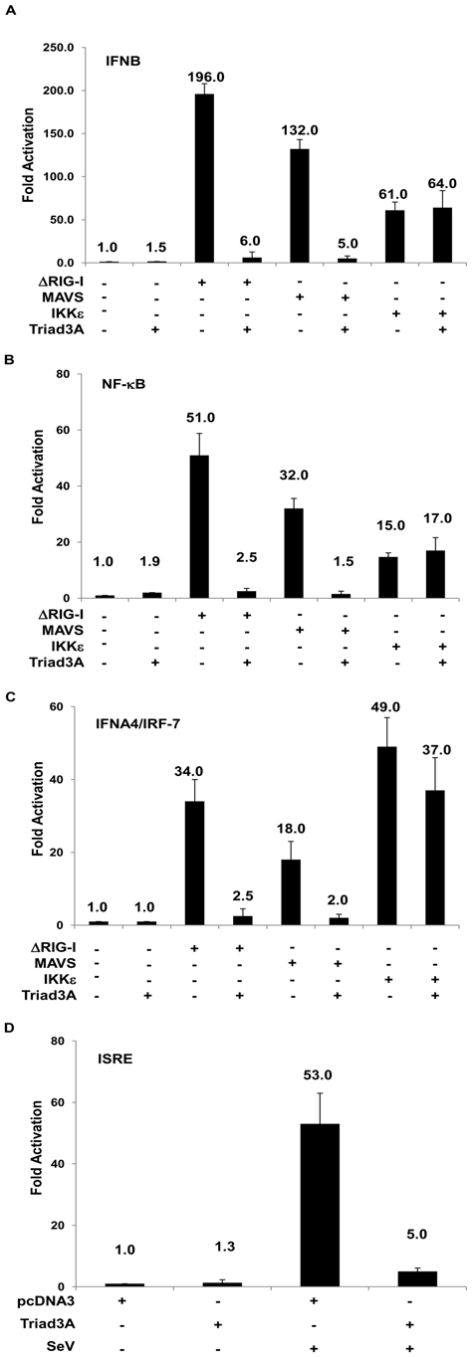
Triad3A inhibits RIG-I/MAVS-mediated transactivation of type 1 IFN promoters. 293T cells were transfected with pRLTK control plasmid and (A) *IFNB*-pGL3, (B), *NF-κB*, (C) *IFNA4*-pGL3 reporter plasmids, the pcDNA3 vector or expression plasmids encoding ΔRIG-I, MAVS, IKKε, as well as Triad3A expression plasmid as indicated. In addition,100 ng of IRF-7 plasmid was added per well for the transactivation of the *IFNA4* promoter. (D) 293T cells were transfected with pcDNA3 or Triad3A expression vector, along with pRLTK control plasmid (100 ng) and ISRE-Luc reporter plasmid (200 ng). 24h post-transfection cells were infected with Sendai virus for 16h. Luciferase activity was analyzed at 24h post-transfection by the Dual-Luciferase Reporter assay as described by the manufacturer (Promega). Relative luciferase activity was measured as fold activation (relative to the basal level of reporter gene in the presence of pcDNA3 vector after normalization with co-transfected RLU activity); values are mean ± S.D. for three experiments.

The identification of a TIM domain in Triad3A prompted us to examine the ability of Triad3A to inhibit RIG-I mediated activation of *IFNB* gene transcription; a constitutively active form of RIG-I (aa 1-229, ΔRIG-I), the MAVS adapter or IKKε, were co-expressed together with Triad3A in 293T cells, together with an *IFNB* promoter luciferase reporter. A low basal activity of the *IFNB* promoter was not affected by Triad3A expression (Figure 1A), while co-expression of ΔRIG-I, MAVS, or IKKε resulted in 196, 132, 61-fold stimulation of the *IFNB* promoter, respectively (Figure 1A). Co-expression of Triad3A with ΔRIG-I or MAVS resulted in a complete inhibition of *IFNB* promoter activity, whereas IKKε mediated activation of the *IFNB* promoter remained unchanged (Figure 1A). Similar results were also obtained with the NF-*κ*B response (Figure 1B); expression of ΔRIG-I, MAVS or IKKε, (co-expressed together with IRF-7) activated *IFNA4* promoter activity 34, 18, 49-fold, respectively, while co-expression of Triad3A blocked *IFNA4* activation (Figure 1C). Furthermore, Triad3A blocked interferon stimulated response element (*ISRE*) activation following Sendai virus infection (Figure 1D). A dose-response curve was performed using the *ISRE* promoter with increasing amounts of Triad3A and ΔRIG-I, MAVS, TRIF, or TBK1 expression plasmids; ΔRIG-I resulted in 893-fold induction of the *ISRE* promoter, and Triad3A co-expression diminished activation in a dose dependent manner (Figure S1A). Similarly, MAVS or TRIF adapters activated the ISRE by 785- and 863-fold, respectively; Triad3A again dramatically reduced ISRE activation (Figure S1B, S1C). In contrast, Triad3A did not significantly decrease TBK1-mediated ISRE activation (Figure S1D). Triad3A co-expression with MDA5 or an active form of TLR3 fused to CD4 (CD4-TLR3) resulted in a complete inhibition of *IFNB* promoter activity (Figure S2A). Triad3A inhibited MDA5-induced *NF-κB* promoter activity; however Triad3A inhibition of CD4-TLR3 mediated *NF-κB* promoter activity was less pronounced (Figure S2B). These experiments suggested that Triad3A was a strong inhibitor of RIG-I signaling to IRF-3, IRF-7 and NF-*κ*B and suggested that Triad3A may target an adapter molecule common to both the TLR and RLR signaling pathways.

### Triad3A inhibits downstream IFN activation

**Figure 2:**
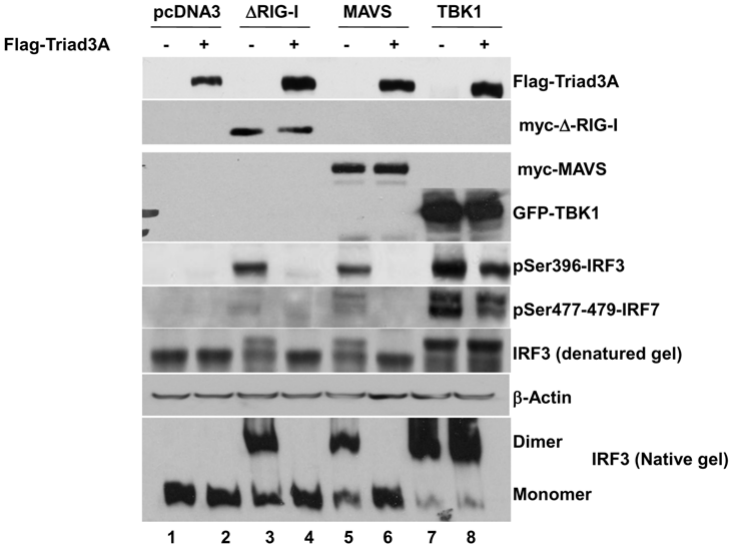
Triad3A inhibits ΔRIG-I and MAVS-mediated activation of IRF-3. 293T cells were cotransfected with 500 ng of IRF-3, 1 µg myc-ΔRIG-I, myc-MAVS, or GFP-TBK1 and 2 µg of Flag-Triad3A expression construct as indicated. Whole cell extracts (40 µg) were resolved by sodium dodecyl sulfate-polyacrylamide gel electrophoresis and analyzed by immunoblotting for IRF-3 pSer396, Flag-Triad3A, myc-ΔRIG-I, myc-MAVS, GFP-TBK1, and β-Actin. For IRF-3 dimerization assay, 293T cells were transfected with 1 µg of pcDNA3-, ΔRIG-I-, MAVS-, or TBK1-expressing plasmid together with 2 µg of pcDNA3 or Triad3A-expressing plasmid as indicated above the lanes. Whole cell extracts (40 µg) were subjected to SDS-PAGE or native PAGE and probed with anti-IRF-3 Ser-396 phosphospecific antibody to detect IRF-3 phosphorylation or anti-IRF-3 antibody to detect IRF-3 dimerization.

As a measure of activation of the IFN signaling pathway, the phosphorylation state of IRF-3 was evaluated by immunoblot in the presence of Triad3A using the phosphospecific Ser-396 IRF-3 antibody [42]. ΔRIG-I co-expression induced Ser-396 IRF-3 phosphorylation (Figure 2, *lane 3*), while co-expression of Triad3A completely blocked IRF-3 phosphorylation (Figure 2, *lane 4*). MAVS expression likewise induced Ser-396 IRF-3 phosphorylation (Figure 2, *lanes 3–5*); that was abrogated by Triad3A (Figure 2, *lanes 4–6*). In contrast, TBK1 co-expression in the presence or absence of Triad3A did not alter the IRF-3 phosphorylation state (Figure 2, *lanes 7–8*). Complementing the phosphorylation status, Triad3A also inhibited ΔRIG-I and MAVS-induced dimerization of endogenous IRF-3 (Figure 2, *lanes 4–6*), but did not affect TBK1-induced IRF-3 dimer formation (Figure 2, *lanes 7–8*), indicating that Triad3A targets RLR signaling upstream of TBK1.

### Triad3A is induced by RNA virus infection and regulates TRAF3 levels

**Figure 3:**
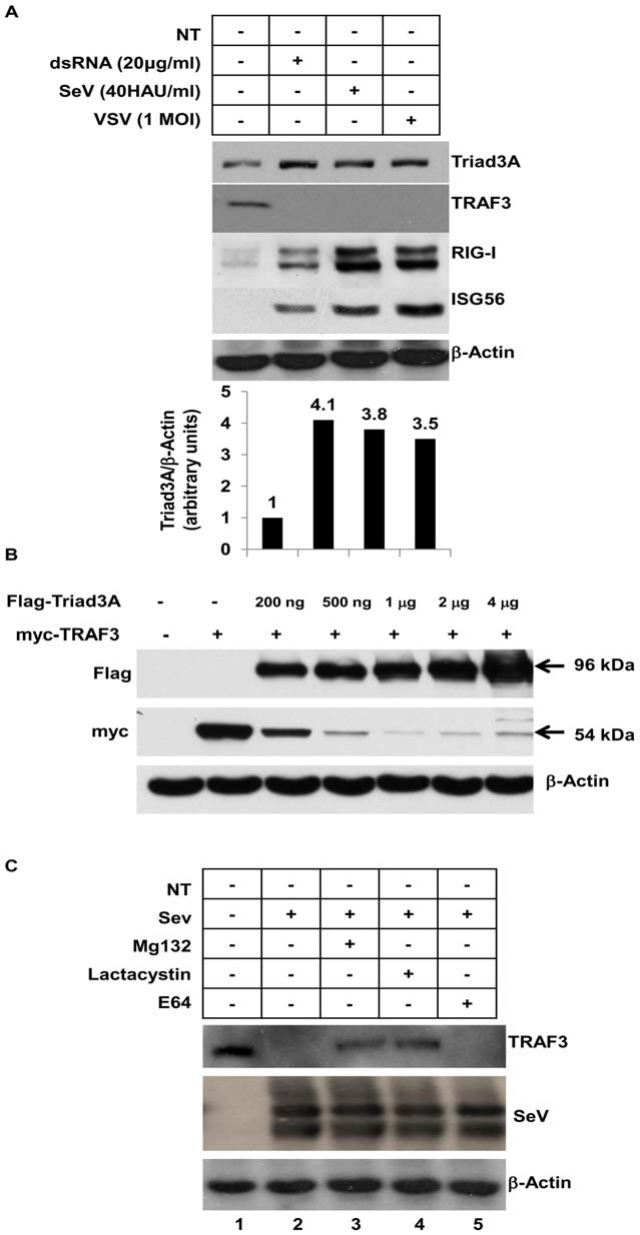
Triad3A is induced by RNA virus infection and regulates TRAF3 levels. (A) A549 cells were treated with 20 µg/ml dsRNA for 6h, or infected with VSV at a MOI 1 or infected with Sendai Virus for 16h. Whole cell extract (40 µg) was resolved by SDS-7.5% PAGE and transferred to nitrocellulose and probed with anti-Triad3A, anti-TRAF3, anti-ISG56, anti-RIG-I and anti-β-Actin antibodies. (B) 293T cells were co-transfected with expression vectors for myc-tagged TRAF3 and increasing amount of expression vector for Flag-tagged Triad3A as indicated. The cells were subsequently lysed, and cell lysates were resolved by SDS-PAGE. The expression levels of TRAF3, Triad3A, and β-Actin were analyzed by immunoblotting with antibodies against myc, Flag, or β-Actin, respectively. (C) A549 cells were infected with Sendai virus 40 HAU/ml and treated with either 5µM of lactacystin or 10µM of Mg132 or 5µM of E64. Whole cell extracts were resolved by SDS-7.5% PAGE and transferred to nitrocellulose and probed with anti-TRAF3, and anti-β-Actin antibodies.

Previous studies demonstrated that the E3 ligase RNF125−a negative regulator of RIG-I− was induced following IFN-α and poly(I∶C) treatment [43]. Endogenous Triad3A protein was induced in human bronchial epithelial A549 cells following dsRNA treatment for 6h, vesicular stomatitis virus (VSV), or Sendai virus (SeV) infection for 16h; correlating with the degradation of TRAF3 protein (Figure 3A). Moreover, Triad3A protein expression is induced following IFN-α/β treatment (data not shown). In addition, it was determined by time-course analysis that 6h dsRNA treatment and 16h virus infection resulted in maximal TRAF3 degradation (Figure S3). Expression of increasing amounts of Triad3A decreased TRAF3 levels in a dose-dependent manner (Figure 3B). Additionally, SeV-mediated degradation of TRAF3 in A549 cells was blocked by the proteasome inhibitors lactacystin and Mg132, but not by the lysosomal protease inhibitor E64 (Figure 3C).

### Stable knock-down of Triad3A correlates with increased TRAF3 protein levels and ISG expression following virus infection

**Figure 4:**
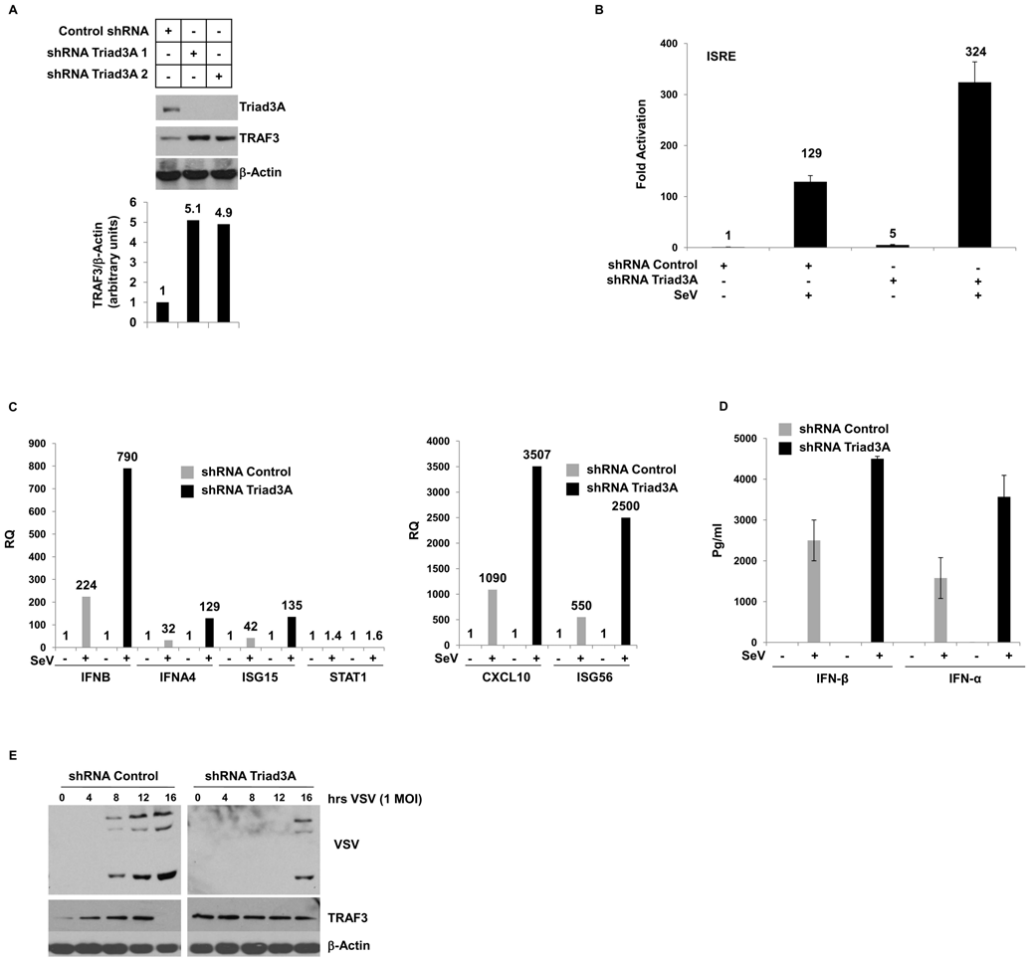
Stable knock-down of Triad3A increases TRAF3 protein levels and ISG expression following virus infection. (A) Whole cell extracts from A549 stable shRNA Triad3A and shRNA control cells was resolved by SDS-7.5% PAGE, transferred to nitrocellulose and probed with anti-Triad3A, anti-TRAF3, and anti-β-Actin antibodies. (B) Stable shRNA Triad3A and control A549 cells were transfected with pRLTK control plasmid (100 ng) and *ISRE*-Luc reporter plasmid (200 ng). Approximately 24h after transfection cells were infected with Sendai virus for 16h as indicated. Luciferase activity was analyzed by the Dual-Luciferase Reporter assay as described by the manufacturer (Promega). Relative luciferase activity was measured as fold activation (relative to the basal level of reporter gene in the presence of pcDNA3 vector after normalization with co-transfected RLU activity); values are mean ± S.D. for three experiments. (C) A549 stable shRNA Triad3A and shRNA control cells were infected with Sendai virus at 40 HAU/ml from 0 to 12h p.i. DNase-treated total RNA were prepared at the indicated times and subjected to real-time PCR analysis for quantification of *IFNB*, *IFNA*, *CXCL10*, *ISG56*, *ISG15*, and *STAT1*. Results are presented as a relative quantification based on the relative expression levels of target gene mRNA versus *B-Actin* mRNA, as a reference gene (values of ratios are indicated on the bar graphs). Normalization using *GAPDH* mRNA levels as reference gave similar results (data not shown). (D) Supernatants from A549 stable shRNA Triad3A and shRNA control cells infected with Sendai virus at 40 HAU/ml were collected 14h post-infection and ELISA assay was performed for IFN-β and IFN-α. (E) A549 stable shRNA Triad3A and shRNA control cells were infected with VSV at a MOI 1 from 0 to 16h p.i. Whole cell extract (40 µg) was resolved by SDS-7.5% PAGE and transferred to nitrocellulose and probed with anti-VSV and anti-TRAF3.

To further confirm the involvement of Triad3A in regulating TRAF3 turnover, two shRNA expression vectors - shRNA1 and shRNA2 that target Triad3A nucleotide sequences 1,532–1,551 and 1,195–1,214, respectively – were used to stably knock-down Triad3A in A549 cells. Knock-down of Triad3A resulted in a 5-fold increase in TRAF3 protein levels (Figure 4A). Interference with endogenous Triad3A also modulated the *ISRE* promoter; ISRE activity was 3-fold higher in Triad3A knock-down cells infected with SeV, compared to cells expressing scrambled shRNA (Figure 4B). [43]. To investigate the physiological effects of Triad3A inhibition on downstream IFN-stimulated target genes, expression of multiple ISGs were examined by quantitative PCR in A549-Triad3A knock-down cells. SeV infection (40 hemagglutination units/ml (HAU)) in Triad3A knockdown cells were led to a 3–4 fold increase in *IFN-β* and *IFN-α2* mRNA expression 12h post-infection (p.i.) compared to control cells (Figure 4C). Similarly, *IP-10 ISG56*, *IS15* transcripts were increased 3–4 fold at 12h p.i. (Figure 4C), while *STAT1* levels remained relatively constant (Figure 4C). In addition, levels of IFN-α and IFN-β released in the supernatant monitored by ELISA increased 2-fold following SeV infection (Figure 4D). Finally, in VSV infected A549 cells, VSV proteins (nucleocapsid (N), surface glycoprotein (G), and matrix (M)) were detected at 8h p.i., whereas in Triad3A knock-down cells, VSV protein expression was delayed, with viral proteins detected only at 16h post-infection (Figure 4E). Notably, in A549 control cells TRAF3 protein levels decreased over time following virus infection, whereas in Triad3A knock-down cells TRAF3 protein levels remained constant (Figure 4E). These results indicate the involvement of Triad3A in regulating IFN and NF-*κ*B dependent gene expression following RNA virus infection.

### TIM domain of Triad3A interacts with the TRAF domain of TRAF3

**Figure 5:**
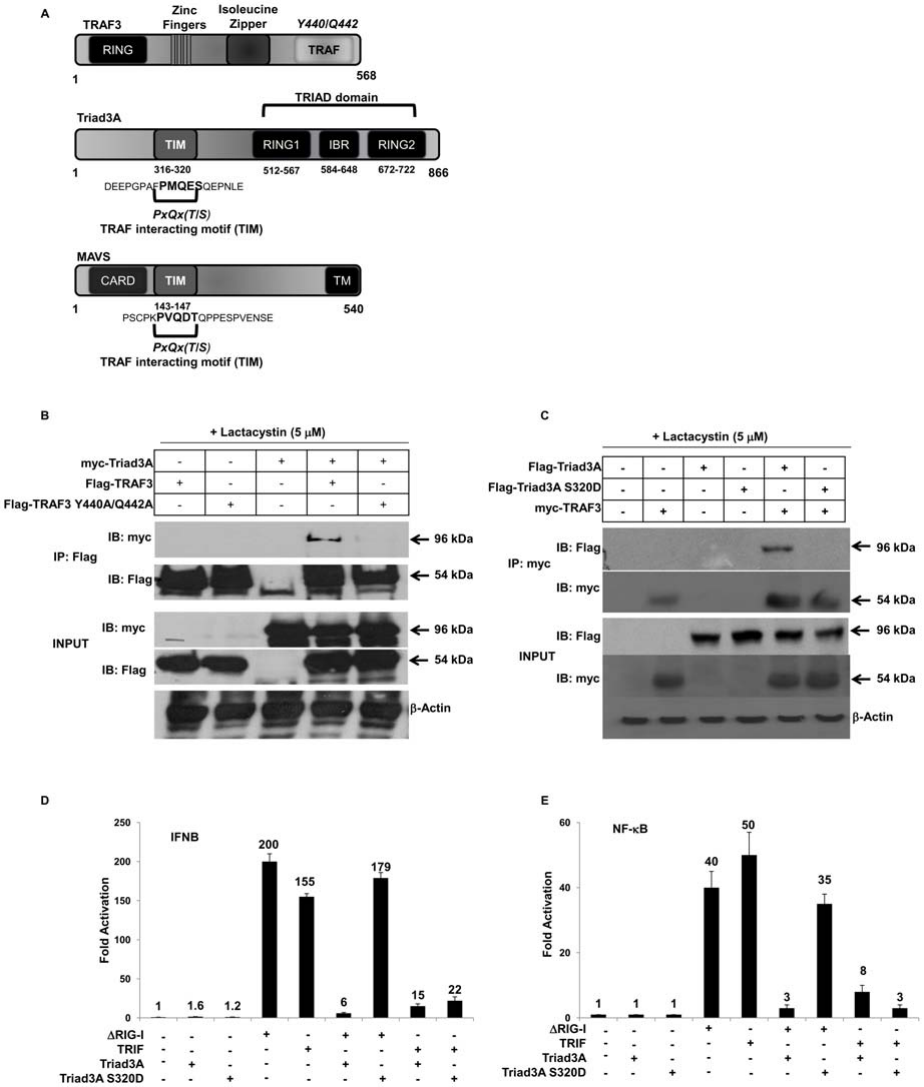
The TIM domain of Triad3A interacts with the TRAF domain. (A) Schematic representation of TRAF3, Triad3A, and MAVS. TRAF3 contains a N-terminus RING domain and a C-terminus TRAF domain where a hydrophobic binding cleft (Y440/Q442) is located. Triad3A contains a TRIAD domain consisting of two RING domains and an ‘in-between-RING’ (IBR) domain. The N-terminus of Triad3A contains a motif (316 -PMQES-320) that matches the consensus TRAF-intercating-motif (TIM), PxQx(T/S). MAVS adapter is composed of a N-terminus CARD domain, as well as a TIM (143-PVQDT-147) located in the proline-rich region. In addition, the C-terminus of MAVS is composed of a Transmembrane (TM) domain for anchoring to the outer-mitochondrial membrane. (B) 293T cells were transfected with myc-tagged Triad3A with Flag-tagged TRAF3 or Flag-TRAF3 Y440A/Q442A as indicated, in the presence of 5µM of Lactacystin at 6h post-transfection. Whole cell extracts were immunoprecipitated with anti-Flag Ab, and then analyzed with anti-myc Ab. (C) 293T cells were transfected with myc-tagged TRAF3 with Flag-tagged Triad3A or Flag-Triad3A S320D as indicated, in the presence of 5µM of Lactacystin at 6h post-transfection. Whole cell extracts were immunoprecipitated with an anti-myc Ab, and then analyzed by with anti-Flag Ab. Cell lysates were analyzed by immunoblotting with anti-myc and anti-Flag antibodies. (D,E) 293T cells were transfected with pRLTK control plasmid, *IFNB*-pGL3 (D), *NF-kB* (E) reporter plasmid and the pcDNA3 vector or expression plasmids encoding ΔRIG-I, TRIF as well as Triad3A or Triad3A S320D expression plasmid as indicated. Luciferase activity was analyzed at 24h post-transfection by the Dual-Luciferase Reporter assay as described by the manufacturer (Promega). Relative luciferase activity was measured as fold activation (relative to the basal level of reporter gene in the presence of pcDNA3 vector after normalization with co-transfected RLU activity); values are mean ± S.D. for three experiments.

The functional specificity of TRAFs is dictated by their ability to recognize and bind distinct structural motifs, termed the TRAF-interacting motif (TIM), with the consensus sequence PxQx(T/S). This motif contacts TRAF proteins within a structurally conserved binding crevice within the C-terminal TRAF domain (Figure 5A). Using multiple sequence alignment, we identified an N-terminal motif in Triad3A - amino acid residues 316 -PMQES- 320 - with substantial homology to the consensus TIM that is also found on the adapter molecule MAVS – amino acid residues 143-PVQDT-147 (Figure 5A). Previously, it has been reported that the TIM domain of MAVS interacts with amino acid residues Y440 and Q442 within the TRAF domain of TRAF3. As a result, co-immunoprecipitation experiments were performed to detect an association of Triad3A and TRAF3; following immunoprecipitation of Flag-tagged TRAF3, immunoblot analysis revealed that TRAF3 and Triad3A co-precipitate together (Figure 5B, *lane 4*). Co-immunoprecipitation of TRAF3 (Y440A/Q442A) and Triad3A revealed that this interaction was impaired, demonstrating that the hydrophobic residues in the TRAF3 binding crevice are important for binding to Triad3A (Figure 5B, *lane 5*). In the reciprocal experiment, Triad3A S320D was unable to bind TRAF3 in co-immunoprecipitation experiments (Figure 5C, *lane 6*) and increasing amounts of Triad3A S320D failed to promote TRAF3 degradation (Figure S4). Furthermore, Triad3A S320D no longer inhibited ΔRIG-I-mediated activation of the *NF-κB* and *IFNβ* gene transcription but readily inhibited TRIF-mediated activation (Figure 5D,E), thus indicating the specificity of the TIM domain of Triad3A for TRAF3.

### Triad3A mediates Lys 48-linked ubiquitination of TRAF3

**Figure 6:**
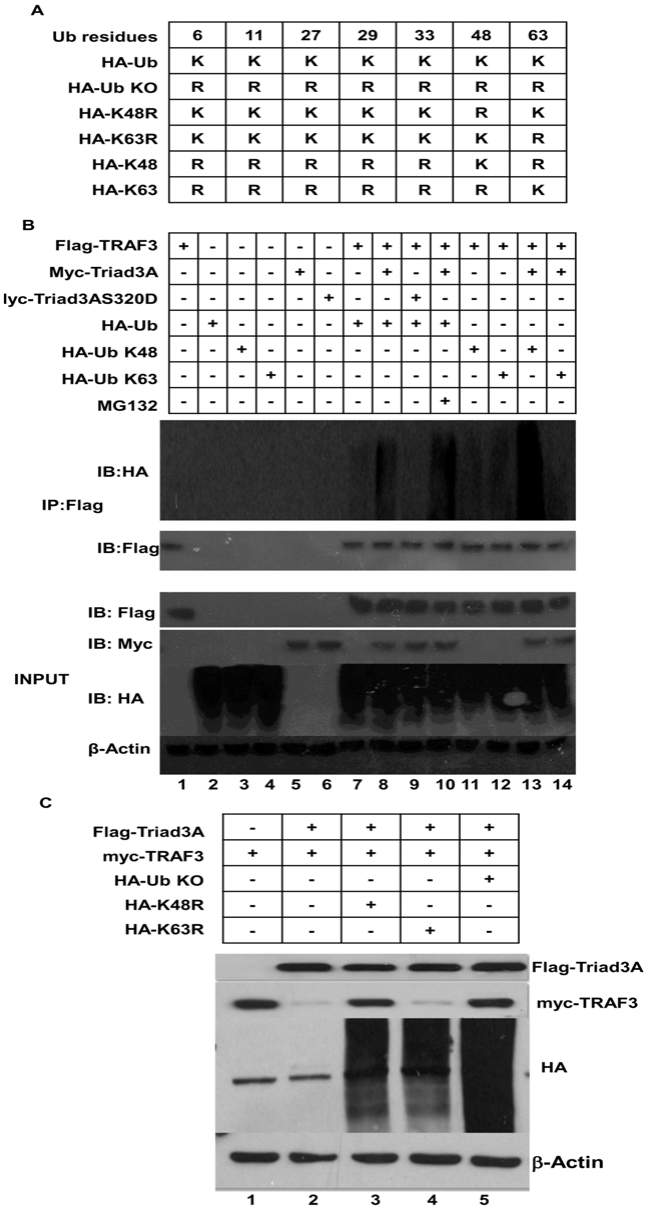
Triad3A promotes Lys^48^-linked polyubiquitination of TRAF3. (A) Schematic illustration of HA- wild type and ubiquitin mutants. (B) 293T cells were transfected with expression vectors for Flag-tagged TRAF3, myc-tagged Triad3A, myc-tagged Triad3AS320D, and wild type or HA-tagged ubiquitin mutants and were treated with 10 µM of Mg132 at 6h post-transfection where indicated. Cell lysates were immunoprecipitated with anti-Flag, and immunoblotted with anti-HA. (C) 293T cells were transfected with Flag-tagged Triad3A, myc-tagged TRAF3, and HA-tagged ubiquitin mutants as indicated.

To test whether Triad3A-mediated degradation of TRAF3 was promoted by Lys^48^-linked ubiquitination, an *in vivo* ubiquitination assay was performed with Flag-tagged TRAF3, HA-tagged wild type or (Lys^48^ and Lys^63^) Ub products (Figure 6A), and sub-optimal levels of myc-tagged Triad3A and Triad3A S320D to limit TRAF3 degradation. Following immunoprecipitation of Flag-tagged TRAF3, immunoblot analysis revealed that Triad3A mediated TRAF3 polyubiquitination (Figure 6B, *lane 8*), with polyubiquitination increasing in the presence of Triad3A and Mg132 (Figure 6B, *lane 10*), compared to TRAF3 and ubiquitin alone (Figure 6B, *lane 7*). In contrast, Triad3A S320D did not polyubiquitinate TRAF3 (Figure 6B, *lane 9*); furthermore, Triad3A promoted Lys^48^-linked polyubiquitination of TRAF3 (Figure 6B, *lane 13*) but not Lys^63^-linked polyubiquitination (Figure 6B, *lane 14*). Cells expressing optimal levels of Triad3A readily degraded TRAF3 (Figure 6C, *lane 2*), whereas Triad3A was unable to degrade TRAF3 in the presence of K48R and KO Ub mutants (Figure 6C, *lane 3,5*).

### TRAF3 undergoes biphasic polyubiquitination and dissociates from MAVS following virus infection

**Figure 7:**
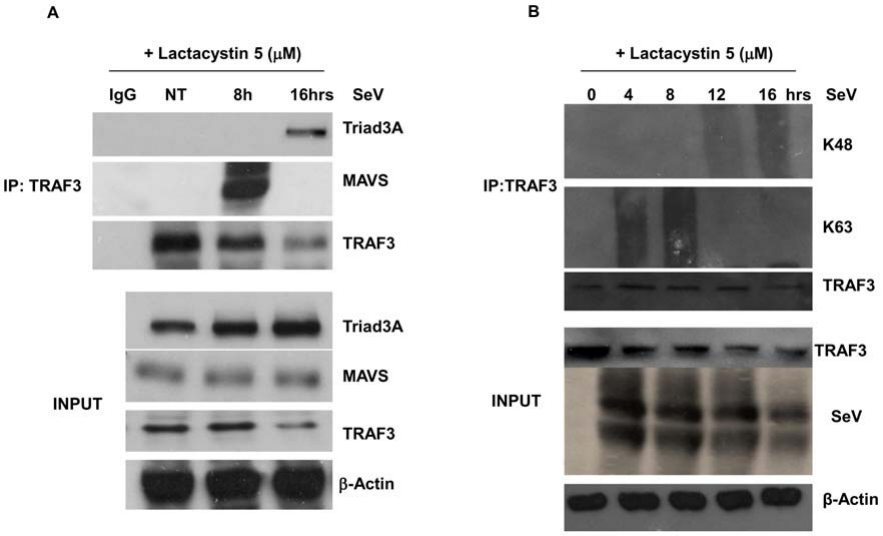
TRAF3 undergoes biphasic polyubiquitination and dissociates from MAVS following virus infection. (A) A549 cells were infected with Sendai virus 40 HAU/ml in the presence of 5µM of Lactacystin, immunoprecipitated with an anti-TRAF3 antibody and then analyzed by immunoblotting with anti-Triad3A and anti-MAVS Ab. (B) A549 cells were infected with Sendai virus 40 HAU/ml in the presence of 5µM of Lactacystin and samples were collected every 4h p.i. Cell lysates were immunoprecipitated with ant-TRAF3, and immunoblotted with Lys^48^ and Lys^63^ anti-ubiquitin specific antibodies.

**Figure 8:**
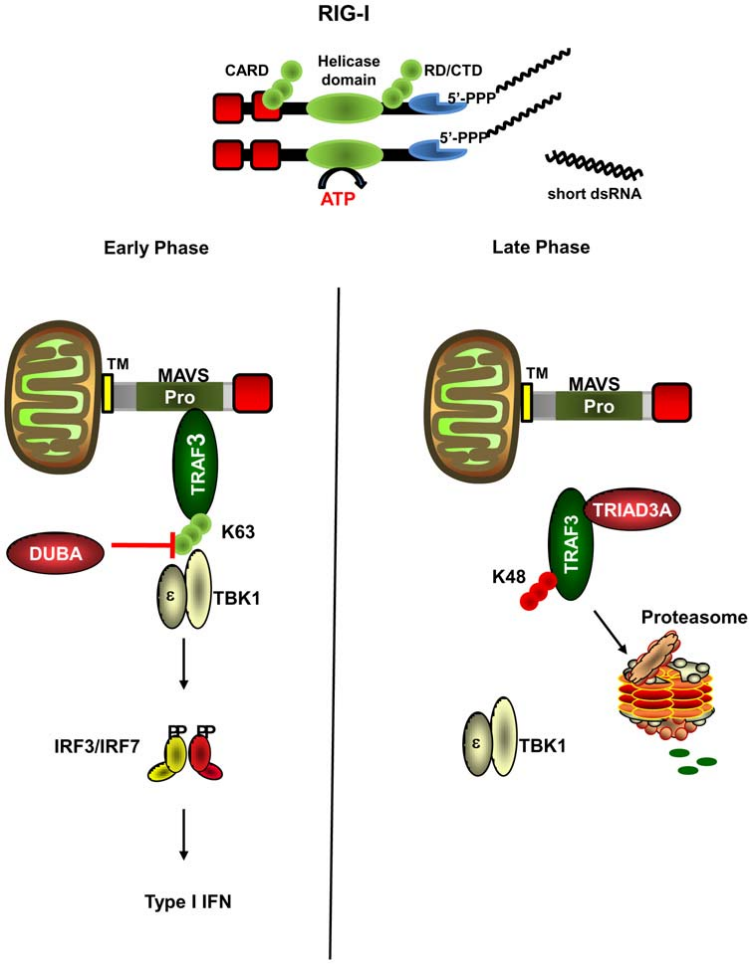
Model of TRAF3 dependent regulation of RIG-I signaling via sequential ubiquitination. At early times after RNA virus infection (4–8h), MAVS interacts with TRAF3 via its TRAF interacting motif (TIM); TRAF3 is subject to Lys^63^ polyubiquitination, leading to recruitment of the downstream kinases TBK1/IKKε and production of type I IFN. Subsequently, DUBA removes Lys^63^ polyubiquitination from TRAF3 which dissociates the TRAF3-TBK1 signaling complex. At late times after infection (12–16h), Triad3A physically associates TRAF3 via its TIM which promotes Lys^48^ polyubiquitination of TRAF3, and subsequent proteasomal degradation.

As both MAVS and Triad3A contain well-characterized TIM domains, the interaction between endogenous TRAF3 and Triad3A was next examined in SeV-infected A549 cells. Following co-immunoprecipitation with anti-TRAF3 antibody, a MAVS-TRAF3 complex was detected at 8h p.i., whereas at 16h, Triad3A disrupted this interaction by associating directly with TRAF3, suggesting that both Triad3A and MAVS compete for the same binding residues on TRAF3 (Figure 7A). Importantly, a kinetic analysis of *in vivo* TRAF3 ubiquitination demonstrated that endogenous TRAF3 was subject to differential biphasic polyubiquitination; using Lys^48^ and Lys^63^ specific Ub antibodies [44], early Lys^63^-linked polyubiquitination was detected at 4h and 8h p.i. (Figure 7B), whereas a late phase Lys^48^-linked polyubiquitination of TRAF3 was detected at 12h and 16h p.i. (Figure 7B). Thus, TRAF3- mediated antiviral signaling appears to be regulated by recruitment of TRAF3 to the MAVS TIM, followed by Triad3A competition for the same binding crevice of TRAF3 (Figure 8).

## Discussion

The present study demonstrates that the E3 ubiquitin ligase Triad3A blocks RIG-I-mediated signaling to NF-κB and IRF pathways by targeting the TRAF3 adapter for degradation via Lys^48^-linked ubiquitinination. Several observations support this conclusion: 1) co-expression of Triad3A blocked ΔRIG-I dependent IRF-3 phosphorylation and dimerization; 2) Triad3A expression decreased TRAF3 protein levels in a dose-dependent manner; 3) knock-down of Triad3A by shRNA increased endogenous TRAF3 protein levels, increased ISG mRNA levels following virus infection, and inhibited VSV replication; 4) Lys^48^-linked ubiquitination of TRAF3 by Triad3A increased TRAF3 turnover; and 5) Triad3A and TRAF3 physically interacted together, an interaction that was impaired by mutation of TRAF3 (Y440A/Q442A), or reciprocally by point mutation of the TIM domain in Triad3A (S320D). TRAF3 appears to undergo a biphasic ubiquitination following virus infection that is crucial for regulation of RIG-I dependent signaling to the antiviral response. Early Lys^63^-linked polyubiquitination of TRAF3 leads to the recruitment of TBK1/IKKε and subsequent activation of the antiviral response [28], while late phase Lys^48^-linked polyubiquitination by Triad3A ultimately degrades TRAF3 and leads to shut-down of the antiviral response (Figure 8).

Recent studies have highlighted the importance of ubiquitination in modulating the innate immune response to invading pathogens via both the TLR and RLR pathways. For example, the RIG-I cytoplasmic RNA sensor undergoes both Lys^48^-linked and Lys^63^-linked ubiquitination [43],[45]: the second CARD domain undergoes TRIM25α-mediated, Lys^63^-linked ubiquitination at Lys-172, resulting in RIG-I/MAVS association and triggering of the antiviral response [45]; RIG-I also undergoes Lys^48^-linked ubiquitination, leading to RIG-I proteasomal degradation by RNF125 [43]. Additionally, RNF125 conjugates ubiquitin to MDA5 and MAVS, thus inhibiting the assembly of the downstream antiviral signaling complex [43]. Overall, multiple steps in the RLR pathway are regulated by ubiquitination to ensure a properly modulated antiviral cascade.

In addition to the newly described role of Triad3A in the regulation of the RIG-I response, previous studies demonstrated that Triad3A negatively regulates both the TLR and TNF-α pathways by promoting Lys^48^-linked, ubiquitin-mediated degradation of TLR4, TLR9 and TIR domain-containing adapters TRIF and TRAM [40],[41]. Triad3A regulation of the TNF-α pathway is achieved via a proteolysis-independent mechanism that impedes RIP1 binding to the TNF-R1 [40],[41]. Furthermore, Triad3A promotes ubiquitination and proteasomal degradation of RIP1 following disruption of the RIP-1-Hsp90 complex. Both Hsp90 and Triad3A form a complex that co-ordinates the homeostasis of RIP1; treatment of cells with geldanamycin to disrupt the Hsp90 complex leads to proteasomal degradation of RIP1 by Triad3A [40]. The present study further illustrates the versatility of Triad3A as a negative regulator of innate signaling pathways.

Both TLR and RLR pathways converge upon TRAF3 in the activation of the antiviral cascade. TRAF3 was originally described as a cytoplasmic adapter that interacted with CD40 and LMP1 and modulated the adaptive immune response [46],[47]. The generation of TRAF3 −/− bone marrow-derived macrophages established TRAF3 as a key molecule in signaling to the production of type I IFNs that functioned as a bridge between MAVS and the downstream kinases TBK1/IKKε [32],[39]. Triad3A mediated degradation of TRAF3 results not only in the inhibition of RIG-I signaling, but also inhibition of MDA5 and TLR3 signaling (Figure S2A, B).

The TIM sequence of MAVS (aa 143-PVQDT-147) binds to the hydrophobic C-terminal crevice of TRAF3 (TRAF domain) located between amino acids Y440 and Q442 [39]. The TIM motif represents a binding interface that recognizes different TRAFs with varying degrees of specificity. The binding cleft in TRAF3 has structurally adaptive “hot spots” that can recognize motifs that are divergent from the consensus TIM [36]. Interestingly, Triad3A interaction with TRAF3 was impaired by mutation of residues within the binding crevice (Y440A/Q442A) (Figure 6B). Furthermore, Triad3A disrupts the interaction between MAVS and TRAF3 (Figure 7A), thus highlighting the importance of the TIM domain of Triad3A in regulating TRAF3 interactions by competitive binding.

In contrast to its positive role in the production of type I IFN, TRAF3 negatively regulates noncanonical p100/p52 NF-*κ*B activation through degradation of the NF-*κ*B inducing kinase NIK [48],[49]. In the present study, co-expression of Triad3A decreased *IFNB*, *IFNA4*, and NF-*κ*B promoter activity by targeting TRAF3 for degradation. Although it was expected that Triad3A driven TRAF3 degradation would enhance NF-*κ*B promoter activity, the observed decrease in NF-κB activity suggests that Triad3A may disrupt other TRAF family members such as TRAF2 and TRAF6, prevent their association with MAVS, and thus disrupt NF-κB activation. However, it has been previously demonstrated that Triad3A does not target TRAF2 or TRAF6 for proteasomal degradation [41]. It is also possible that some components of the p100/p52 pathway may be engaged downstream of RIG-I; this idea is strengthened by the recent report that TNFR1-associated death domain protein (TRADD) is essential for RIG-I/MAVS signaling, forms a complex with TRAF3/TANK/FADD/RIP1, and leads to activation of IRF-3 and NF-*κ*B [50]. Furthermore, the effect of Triad3A on NF-*κ*B activation was shown to be independent of RIP1 proteolytic degradation [41], thus strengthening the possibility that another TRAF family member associates with the TIM domain of Triad3A.

Previous studies demonstrated that TRAF3 signaling was tightly regulated by the de-ubiquitinase A (DUBA) which removed Lys^63^ linked Ub residues from TRAF3 and disrupted recruitment of TBK1/IKKε and downstream IFN activation [28]. Dual regulation of TRAF3 by DUBA and Triad3A represents a pivotal point in the control of RLR signaling. The present results suggest a biphasic regulation or “immune-editing”, whereby TRAF3 is Lys^63^ polyubiquitinated early after virus infection to bridge protein-protein interactions between MAVS and TBK1/IKKε. Later, Lys^63^ polyubiquitin is removed by DUBA to disrupt TRAF3-TBK1/IKKε interactions [28]; TRAF3 then undergoes a late phase Lys^48^-linked polyubiquitination by Triad3A, leading to proteasomal degradation (Figure 8). Such a multi-level regulation of TRAF3 underscores its key role in modulating positive and negative antiviral signaling. Furthermore, the complementary functions of DUBA and Triad3A with respect to inhibition of TRAF3 activity and turnover may be subject to stimuli- and tissue-specific regulation, a topic that warrants further investigation. In conclusion, Triad3A acts as a multi-targeting E3 ubiquitin ligase that negatively regulates the TLR, TNF-α and RLR pathways; in the RLR pathway, Triad3A targets TRAF3 for Lys^48^-linked polyubiquitination, leading to proteasome-dependent degradation, as part of the host-specific mechanism that limits the antiviral response.

## Materials and Methods

### Plasmid constructions and mutagenesis

Plasmids encoding ΔRIG-I, MAVS, IKKε, TBK1, *NF-κB/*pGL3, *IFNB*/pGL3, *IFNA4*/pGL3, *ISRE*-luc reporter, and pRLTK were described previously [14],[24],[51],[52]. HA-ubiquitin and other HA-Ubiquitin constructs (HA-Ub-K48, HA-Ub-K63, HA-Ub-K48R, HA-Ub-K63R, and HA-Ub-KO) were kind gifts from Dr. Zhijian Chen (Department of Molecular Biology, University of Texas Southwestern Medical Center, Dallas Texas). MDA5 and CD4-TLR3 were kind gifts from Dr. Stephen Goodbourn (Division of Basic Medical Sciences, St George's, University of London, England) and Dr. Luke A. J. O'Neill (School of Biochemistry and Immunology, Trinity College, Dublin, Ireland) respectively. Human Triad3A cDNA was amplified from pKR5 Flag-Triad3A expression plasmid and cloned into Flag and myc pcDNA3.1/Zeo. The Triad3A point mutant S320D was introduced by Quickchange Kit according to the manufacturer's instructions (Stratagene). DNA sequencing was performed to confirm the mutation. Triad3A shRNA1 targeting nucleotide sequence (1,532–1,551) 5′-GAGCAGGAGTTCTATGAGCA-3′, shRNA2 targeting nucleotide sequence (1,195–1,214) 5′-GGACACTATGCAATCACCCG-3′ and shRNA control have been previously described [40]. Human TRAF3 cDNA was amplified from pKR5 Flag-TRAF3 and pKR5 Flag-TRAF3 Y440A/Q442A expression plasmids provided by Dr. Genhong Cheng (UCLA, USA) and were cloned into Flag pcDNA3.1/Zeo. Mg132, lactacystin and E64 were purchased from Calbiochem. dsRNA was purchased from Invivogen. A549 cells were infected with Sendai virus (40 HAU/ml) for 16h and were treated with either Mg132 (10µM), lactacystin (5µM) or E64 (5µM) 6h p.i.

### Cell culture, transfections, and luciferase assays

Transfections for Luciferase assay were carried out in 293T cells grown in Dulbecco's modified Eagle's medium (Invitrogen) supplemented with 10% fetal bovine serum and antibiotics. Subconfluent 293T cells were transfected with 100 ng of pRLTK reporter (*Renilla* luciferase for internal control), 200 ng of pGL-3 reporter (firefly luciferase, experimental reporter), 200 ng of ΔRIG-I, MDA5, CD4-TLR3, MAVS, TRIF, IKKε, or TBK1 expression plasmids, 200 ng of pcDNA3 or Flag Triad3A/Flag Triad3A S320D pcDNA3, and 100ng of IRF-7 plasmid as indicated by calcium phosphate co-precipitation method. The reporter plasmids were: *IFNB* pGL3, *ISRE*-luc, *NF-κB* pGL3, and *IFNA4* pGL-3 reporter genes; the transfection procedures were previously described [53]. At 24h after transfection, the reporter gene activities were measured by Dual-Luciferase Reporter Assay, according to manufacturer's instructions (Promega). Where indicated, cells were treated with Sendai virus (40 HAU/ml) for the indicated time or 16h for luciferase assays. Human A549 cells were cultured in F12K medium (Wisent Inc.) supplemented with 10% fetal bovine serum, glutamine and antibiotics. A549 cells were transfected either with dsRNA (20µg/ml) for 6h or infected with VSV-AV1 (multiplicity of infection of 1 (MOI)) for 16h or Sendai virus (40 HAU/ml) for 16h.

### Generation of Triad3A knock-down cells

shRNA1 Triad3A and shRNA Control were transfected into A549 cells by using the Fugene 6 transfection reagent (Roche Applied Sciences). Cells were selected beginning at 48h post-transfection for 3 weeks in Dulbecco's modified Eagle's medium containing 10% heat-inactivated calf serum, glutamine, antibiotics, and 2 µg/ml G418 (Invitrogen); individual clones were screened for maximal knockdown of Triad3A by immunoblot.

### In vivo ubiquitination assay

293T cells were transiently transfected with 2.5 µg Flag-TRAF3, 250 ng myc-Triad3A, 250 ng myc-Triad3A S320D and 1 µg HA-Ubiquitin expression plasmids. At 6h post-transfection, cells were treated with 10 µM of Mg132 where indicated. Samples were harvested 24h post-transfection, lysed using a 1% NP-40 lysis buffer (50 mM Tris-HCL ph 7.5, 150 mM NaCl, 5mM EDTA, 50 mM NaF, 1% NP-40, 10% glycerol, 30 mMβ-glycerophosphate, 1mM orthovanadate (Na_3_VO_4_), 1 mM phenyl-methyl-sulfonyl fluoride (PMSF)) supplemented with 0.1% protease inhibitor cocktail (Sigma-Aldrich, Oakville, Ont.) and the deubiquitinase inhibitor N-ethylmaleimide (NEM, 10 mM, Sigma-Aldrich, Oakville, Ont). Samples were boiled for 10 minutes in 1% SDS and diluted 10 times in lysis buffer. 250 µg of proteins were then immunoprecipitated overnight at 4°C with constant agitation with 0.5 µg of anti-Flag (M2; Sigma-Aldrich) crosslinked to 30 µl of protein A/G PLUS-Agarose (Santa Cruz Biotechnology). Immunoprecipitated protein was washed 4 times with supplemented lysis buffer, denatured in 2% SDS-loading dye, and loaded onto a 7.5% acrylamide gel for SDS-PAGE analysis followed by transfer to nitrocellulose membrane. Polyubiquitination was detected by immunoblotting with a monoclonal anti-HA antibody (Sigma-Aldrich, Oakville, Canada). A549 cells were infected with Sendai virus (40 HAU/ml) in the presence of 5 µM of lactacystin and samples were collected every 4h p.i. Samples were lysed as previously described and samples were boiled for 10 minutes in 1% SDS and diluted 10 times in lysis buffer. 500 µg of proteins were then immunoprecipitated overnight at 4°C with constant agitation with 0.5 µg of anti-TRAF3 (sc-6933 Santa Cruz, USA) crosslinked to 30 µl of protein A/G PLUS-Agarose (Santa Cruz Biotechnology). Immunoprecipitated protein was washed 4 times with supplemented lysis buffer, denatured in 2% SDS-loading dye, and loaded onto a 7.5% acrylamide gel for SDS-PAGE analysis followed by transfer to nitrocellulose membrane. Polyubiquitination was detected by immunoblotting with polyclonal Lys^48^ and Lys^63^ anti-ubiquitin specific antibodies (Millipore, USA).

### Co-immunoprecipitation

Cells were lysed in lysis buffer (50 mM Tris-HCl, pH 7.5, 250 mM NaCl, 0.5% NP-40) supplemented with 0.1% protease inhibitor cocktail (Sigma-Aldrich, Oakville, Canada). 250 µg of proteins were then immunoprecipitated overnight at 4°C with constant agitation with either 0.5 µg of anti-myc (9E10; Sigma-Aldrich) or 0.5 µg of anti-Flag (M2; Sigma-Aldrich) or 0.5 ug of anti-TRAF3 crosslinked to 30 µl of protein A/G PLUS-Agarose (Santa Cruz Biotechnology). After extensive washing with lysis buffer, the immunocomplexes were analyzed by immunoblotting as described.

### Immunoblot analysis

Whole cell extracts (20–40 µg) were separated in 7.5–12% acrylamide gel by SDS-PAGE and were transferred to a nitrocellulose membrane (BioRad, Mississauga, Canada) at 4°C for 1h at 100 V in a buffer containing 30 mM Tris, 200 mM glycine and 20% (vol/vol) methanol. Membranes were blocked for 1h at room temperature in 5% (vol/vol) dried milk in PBS and 0.1% (vol/vol) Tween-20 and then were probed with primary antibodies. Anti-Flag (M2), anti-Hemagglutinin HA (H7), or anti-myc (9E10) each at a concentration of 1 µg/ml were purchased from Sigma-Aldrich (Sigma-Aldrich, Oakville, Canada); anti-MAVS (1∶1000, in-house previously described [14]) were prepared in blocking solution plus 0.02% sodium azide. Anti-IRF-3 (1∶5000, IBL, Japan), anti-β-Actin (1∶5000, MAB1501 Millipore, USA), anti-Triad3A (1∶1000, ProSci Inc. USA), anti-RIG-I (1∶1000, rabbit polyclonal Ab, previously described [14]), anti-VSV (1∶3000, rabbit polyclonal Ab raised against VSV proteins G, N, and M), anti-ISG56 (1∶1000, gift from Dr. Ganes Sen, Cleveland Clinic), anti-TRAF3 (1 µg/ml, sc-6933 Santa Cruz, Cal, USA), anti-IRF-3 Ser 396 (1∶1000, rabbit anti-peptide Ab, previously described [54]), and Lys^48^ and Lys^63^ anti-ubiquitin specific antibody (1∶1000, Millipore, USA) were prepared in 3% BSA/PBS/0.03% sodium azide.

### IRF-3 dimerization

Whole cell extracts were prepared in Nonidet P-40 lysis buffer (50 mM Tris, pH 7.4, 150 mM NaCl, 30 mM NaF, 5 mM EDTA, 10% glycerol, 1.0 mM Na_3_VO_4_, 40 mM β-glycerophosphate, 0.1 mM phenylmethylsulfonyl fluoride, 5 µg/ml of each leupeptin, pepstatin, and aprotinin, and 1% Nonidet P-40), and then were subjected to electrophoresis on 7.5% native acrylamide gels, which were pre-run for 30 min at 4°C. The electrophoresis buffers were composed of an upper chamber buffer (25 mM Tris, pH 8.4, 192 mM glycine, and 1% sodium deoxycholate) and a lower chamber buffer (25 mM Tris, pH 8.4, 192 mM glycine). Gels were soaked in SDS running buffer (25 mM Tris, pH 8.4, 250 mM glycine, 0.1% SDS) for 30 min at 25°C and were then electrophoretically transferred on Hybond-C nitrocellulose membranes (Amersham Biosciences) in 25 mM Tris, pH 8.4, 192 mM glycine, and 20% methanol for 1 h at 4°C. Membranes were blocked in phosphate-buffered saline containing 5% (vol/vol) nonfat dry milk and 0.05% (vol/vol) Tween 20 for 1 h at 25°C and then were blotted with an antibody against IRF-3 (1 µg/ml) in blocking solution for 1 h at 25°C. After washing the membranes five times in phosphate-buffered saline/0.05% Tween, they were incubated for 1 h with horseradish peroxidase-conjugated goat anti-rabbit IgG (1∶4000) in blocking solution. Immunoreactive bands were visualized by enhanced chemiluminescence (Amersham Biosciences).

### Real-time PCR

Quantitative PCR assays were performed in triplicate using the AB 7500 Real-time PCR System (Applied Biosystems). The primers used were as follows: *IFN-ß*, 5′-TTGTGCTTCTCCACTACAGC-3′ (forward) and 5′-CTGTAAGTCTGTTAATGAAG-3′ (reverse); *IFN-α2*, 5′-CCTGATGAAGGAGGACTCCATT-3′ (forward) and 5′-AAAAAGGTGAGCTGGCATACG-3′ (reverse); *ISG15*, 5′-AGCTCCATGTCGGTGTCAG-3′ (forward) and 5′-GAAGGTCAGCCAGAACAGGT-3′ (reverse); *ISG56* 5′-CAACCAAGCAAATGTGAGGA-3′ (forward) and 5′-AGGGGAAGCAAAGAAAATGG-3′ (reverse); *CXCL10* 5′-TTCCTGCAAGCCAATTTTGTC-3′ (forward) and 5′-TCTTCTCACCCTTCTTTTTCATTGT-3′ (reverse); *STAT1* 5′-CCTGCTGCGGTTCAGTGA-3′ (forward) and 5′-TCCACCCATGTGAATGTGATG-3′ (reverse); *ß-Actin*, 5′-CCTTCCTGGGCATGGAGTCCT-3′ (forward) and 5′-AATCTCATCTTGTTTTCTGCG-3′ (reverse). All data are presented as a relative quantification with efficiency correction based on the relative expression of target genes versus *ß-Actin* as reference gene. Standard curves and PCR efficiencies were obtained using serial dilutions of pooled cDNA prepared from stable shRNA1 Triad3A and shRNA control A549 cells infected with Sendai virus (40 HAU/ml) for 12h. Data were then collected using the AB 7500 Real-time PCR System (Applied Biosystems) and analyzed by comparative *C*_T_ method using the SDS version 1.3.1 Relative Quantification software.

### ELISA

The supernatants from stable shRNA Triad3A and shRNA Control cells infected with Sendai virus (40 HAU/ml) were collected at 12h p.i. The concentrations of IFN-β and IFN-α in the supernatants were measured using ELISA kits (PBL Biomedical Laboratories, Piscataway, NJ).

## Supporting Information

Figure S1 Triad3A blocks RIG-I/MAVS and TRIF-mediated ISRE transactivation. 293T cells were transfected with pRLTK control plasmid (100 ng), *ISRE*-Luc reporter plasmid (200 ng), RIG-I (A)-, MAVS (B)-, TRIF (C)-, or TBK1(D)-expressing plasmid (200 ng) together with an increase amount of Triad3A expression plasmid (0, 50, 200, and 1000 ng) as indicated. In all transfections, the pcDNA3 vector was added to bring the total plasmids to 1500 ng. Luciferase activity was analyzed at 24h post-transfection by the Dual-Luciferase Reporter assay as described by the manufacturer (Promega). Relative luciferase activity was measured as fold activation (relative to the basal level of reporter gene in the presence of pcDNA3 vector after normalization with co-transfected RLU activity); values are mean ± S.D. for three experiments.
(0.37 MB TIF)

Figure S2 Triad3A inhibits MDA5 and CD4-TLR3 transactivation. 293T cells were transfected with pRLTK control plasmid and *IFNB*-pGL3 (A), *NF-κB* (B) reporter plasmid and the pcDNA3 vector or expression plasmids encoding MDA5 and CD4-TLR3, as well as Triad3A expression plasmid as indicated. Luciferase activity was analyzed at 24h post-transfection by the Dual-Luciferase Reporter assay as described by the manufacturer (Promega). Relative luciferase activity was measured as fold activation (relative to the basal level of reporter gene in the presence of pcDNA3 vector after normalization with co-transfected RLU activity); values are mean ± S.D. for three experiments.
(0.44 MB TIF)

Figure S3 dsRNA treatment promotes TRAF3 degradation. A549 cells were treated with dsRNA 20µg/ml and cells were collected every 2h post-treatment. Cell lysates were analyzed by immunoblotting with anti-TRAF3, anti-SeV, and anti-ISG56 antibodies.
(0.25 MB TIF)

Figure S4 Triad3A S320D does not alter TRAF3 protein expression. 293T cells were co-transfected with expression vectors for Flag-tagged TRAF3 and increasing amount of expression vector for myc-tagged Triad3A S320D as indicated. The cells were subsequently lysed, and cell lysates were resolved by SDS-PAGE. The expression levels of TRAF3, Triad3A S320D, and β-actin were analyzed by immunoblotting with antibodies against myc, Flag, or β-Actin, respectively.
(0.39 MB TIF)
