# Supplementary figures and images for: The E3 Ubiquitin Ligase Triad3A Negatively Regulates the RIG-I/MAVS Signaling Pathway by Targeting TRAF3 for Degradation
Source: PLoS Pathog. 2009 Nov 6;5(11):e1000650. doi: 10.1371/journal.ppat.1000650 (PMC2766052; doi:10.1371/journal.ppat.1000650)

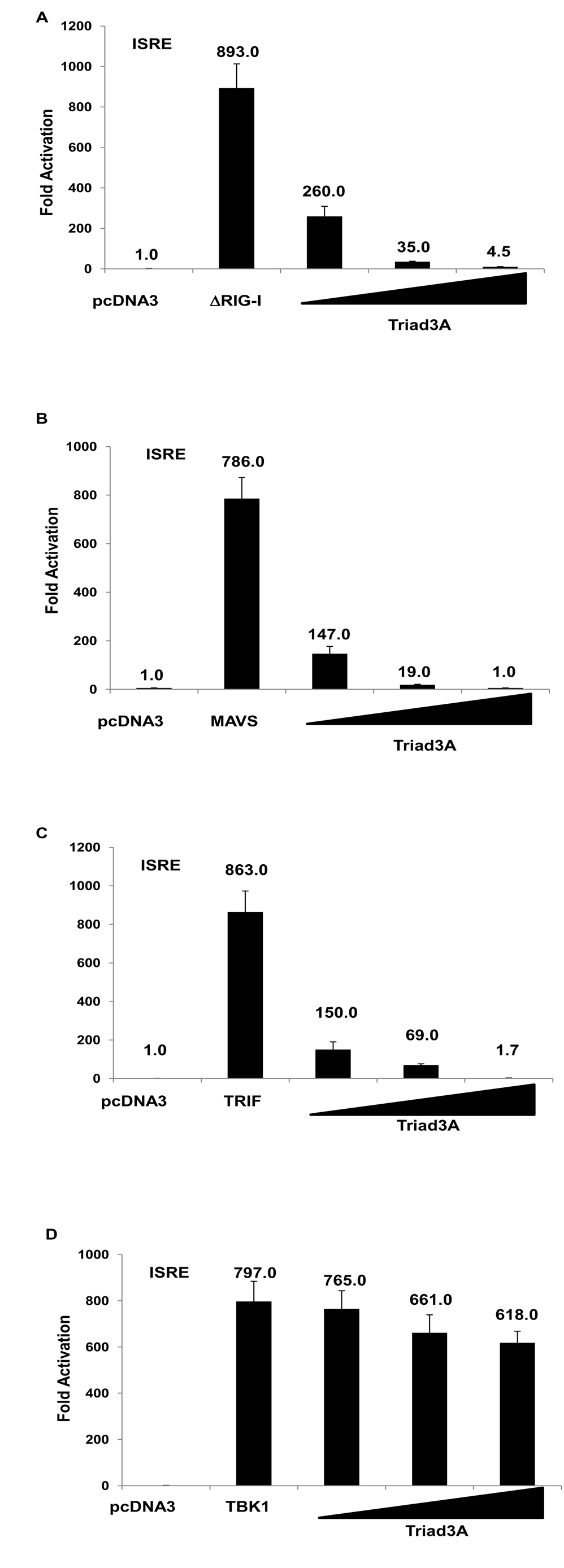

Supplement: Figure S1 — Triad3A blocks RIG-I/MAVS and TRIF-mediated ISRE transactivation. 293T cells were transfected with pRLTK control plasmid (100 ng), ISRE-Luc reporter plasmid (200 ng), RIG-I (A)-, MAVS (B)-, TRIF (C)-, or TBK1(D)-expressing plasmid (200 ng) together with an increase amount of Triad3A expression plasmid (0, 50, 200, and 1000 ng) as indicated. In all transfections, the pcDNA3 vector was added to bring the total plasmids to 1500 ng. Luciferase activity was analyzed at 24h post-transfection by the Dual-Luciferase Reporter assay as described by the manufacturer (Promega). Relative luciferase activity was measured as fold activation (relative to the basal level of reporter gene in the presence of pcDNA3 vector after normalization with co-transfected RLU activity); values are mean ± S.D. for three experiments. (0.37 MB TIF) [file ppat.1000650.s001.tif]

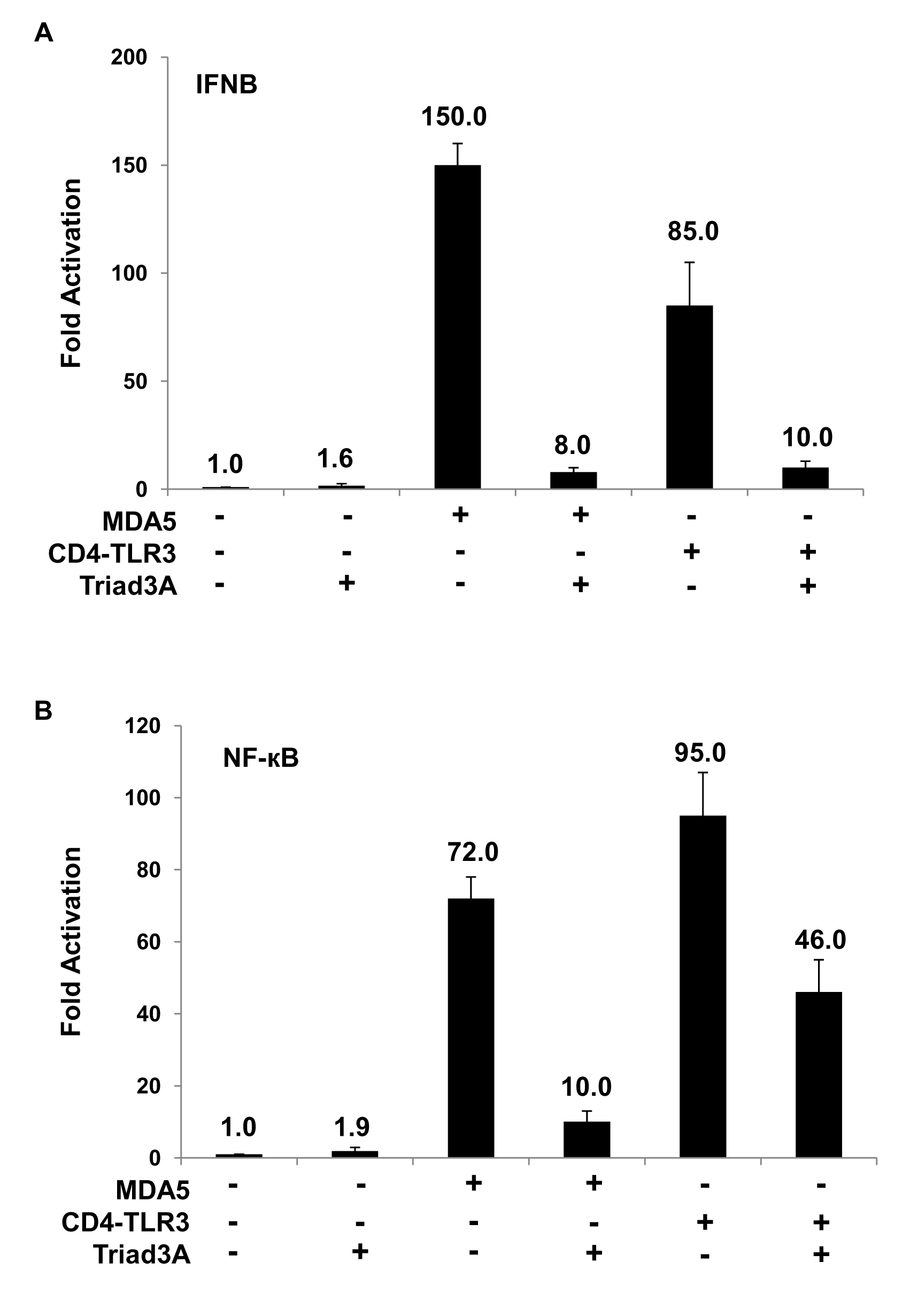

Supplement: Figure S2 — Triad3A inhibits MDA5 and CD4-TLR3 transactivation. 293T cells were transfected with pRLTK control plasmid and IFNB-pGL3 (A), NF-κB (B) reporter plasmid and the pcDNA3 vector or expression plasmids encoding MDA5 and CD4-TLR3, as well as Triad3A expression plasmid as indicated. Luciferase activity was analyzed at 24h post-transfection by the Dual-Luciferase Reporter assay as described by the manufacturer (Promega). Relative luciferase activity was measured as fold activation (relative to the basal level of reporter gene in the presence of pcDNA3 vector after normalization with co-transfected RLU activity); values are mean ± S.D. for three experiments. (0.44 MB TIF) [file ppat.1000650.s002.tif]

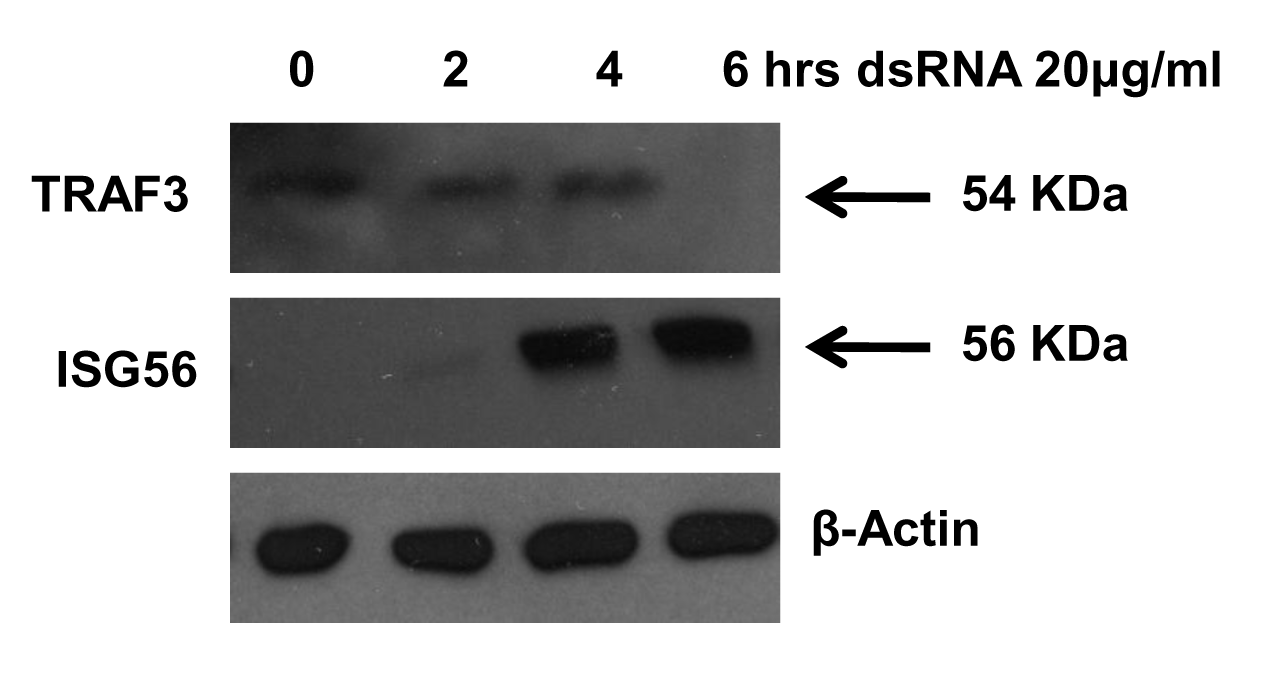

Supplement: Figure S3 — dsRNA treatment promotes TRAF3 degradation. A549 cells were treated with dsRNA 20µg/ml and cells were collected every 2h post-treatment. Cell lysates were analyzed by immunoblotting with anti-TRAF3, anti-SeV, and anti-ISG56 antibodies. (0.25 MB TIF) [file ppat.1000650.s003.tif]

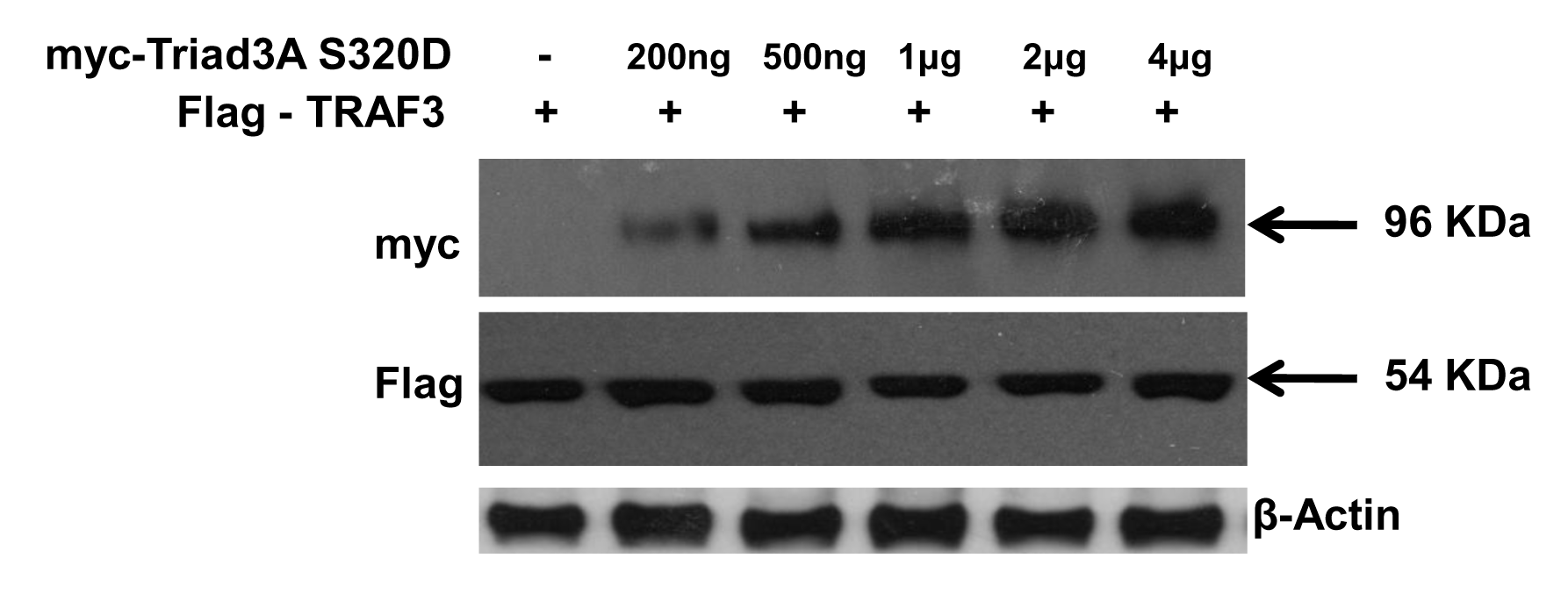

Supplement: Figure S4 — Triad3A S320D does not alter TRAF3 protein expression. 293T cells were co-transfected with expression vectors for Flag-tagged TRAF3 and increasing amount of expression vector for myc-tagged Triad3A S320D as indicated. The cells were subsequently lysed, and cell lysates were resolved by SDS-PAGE. The expression levels of TRAF3, Triad3A S320D, and β-actin were analyzed by immunoblotting with antibodies against myc, Flag, or β-Actin, respectively. (0.39 MB TIF) [file ppat.1000650.s004.tif]
